# Datos sintéticos de un modelo de datos común para las aplicaciones de inteligencia artificial en salud materna: reporte de experiencia en el contexto colombiano

**DOI:** 10.7705/biomedica.7937

**Published:** 2025-12-10

**Authors:** Ever Augusto Torres-Silva, Juan José Gaviria-Jiménez, Ana María Guevara-Zambrano, Laura Herrera-Almanza, José Flórez-Arango

**Affiliations:** 1 Futuro, Netux S.A.S., Medellín, Colombia Netux S.A.S. Futuro Netux S.A.S. Medellín Colombia; 2 Facultad de Medicina, Universidad de Antioquia, Medellín, Colombia Universidad de Antioquia Facultad de Medicina Universidad de Antioquia Medellín Colombia; 3 Ginecología y Obstetricia, Hospital Pablo Tobón Uribe, Medellín, Colombia Hospital Pablo Tobón Uribe Ginecología y Obstetricia Hospital Pablo Tobón Uribe Medellín Colombia; 4 Investigación, Clínica Universitaria Bolivariana, Medellín, Colombia Clínica Universitaria Bolivariana Investigación Clínica Universitaria Bolivariana Medellín Colombia; 5 Population Health Sciences, Weill Cornell Medicine, New York, USA Weill Cornell Medicine Population Health Sciences Weill Cornell Medicine New York USA

**Keywords:** registros electrónicos de salud, salud materna, embarazo, inteligencia artificial., Electronic health records, maternal health, pregnancy, artificial intelligence.

## Abstract

**Introducción.:**

Los datos sintéticos en salud son una alternativa para generar registros clínicos que permitan obtener historias clínicas similares a las reales y que puedan ser usadas en diferentes situaciones clínicas.

**Objetivo.:**

Formular un modelo basado en la generación de datos sintéticos para el proceso de atención de la gestación en Colombia y adaptarlo al modelo de datos común de la *Observational Medical Outcomes Partnership* (OMOP) para facilitar su integración en aplicaciones de inteligencia artificial en salud materna.

**Materiales y métodos.:**

Se realizó un estudio de caso de formulación de datos completamente sintéticos, en el cual se incluyeron algunos de los desenlaces y condiciones más frecuentes de la gestación durante un proceso típico de atención de mujeres gestantes en Colombia. La propuesta se complementó con la generación de un modelo común de datos para facilitar la integración de los datos en futuras aplicaciones de inteligencia artificial o de sistemas complementarios que se beneficien de un lenguaje común, independiente del sistema o de la forma de clasificación.

**Resultados.:**

Se logró la formulación de un modelo para la generación sintética de datos clínicos en el entorno clínico de atención de la gestación hasta el periodo perinatal. El modelo incluyó las condiciones clínicas y los desenlaces más frecuentes, los cuales se diagramaron en la herramienta Synthea™ con sus respectivas probabilidades clínicas de ocurrencia, según la literatura reportada o la práctica habitual de los especialistas en obstetricia en Colombia.

**Conclusiones.:**

Este estudio demuestra que la generación de datos sintéticos aplicados al proceso de atención de la gestación en Colombia es factible y constituye un aporte pionero en la región.

## Abstract (en)

**Introduction.:**

Synthetic data in healthcare is an alternative for generating clinical records that resemble those registered in real clinical scenarios. The benefits of synthetic data are: greater volume of data, the possibility of representing specific patient populations, protection of real-data privacy, and improved data-sharing among different actors.

**Objective.:**

To formulate a synthetic data generation model for the gestational care process in Colombia and adapt it to the Observational Medical Outcomes Partnership (OMOP) common data model to facilitate its integration into artificial intelligence applications in maternal health.

**Materials and methods.:**

We conducted a case study of fully synthetic data formulation that included some of the most frequent outcomes and conditions during gestation based on a typical care process for pregnant women in Colombia. This approach was complemented by the generation of a common data model to facilitate data integration in future artificial intelligence applications or complementary systems that benefit from a standardized language, regardless of the system or form of classification.

**Results.:**

We formulated a model for the synthetic generation of clinical data -applicable to real clinical settings- that spans the entire gestational care until the perinatal period. The model included the most frequent clinical conditions and outcomes, which were diagrammed in the Synthea™ tool with their corresponding clinical probabilities of occurrence based on the reported literature or the usual practice of obstetric specialists in Colombia.

**Conclusions.:**

This study demonstrates that the generation of synthetic data applied to the gestational care process in Colombia was feasible and represents a pioneering contribution in the region.

La informática biomédica es la utilización de datos, información y conocimientos generados en el sector salud para la resolución de problemas y la toma de decisiones ^1^. Esto se ve plasmado en la generación de sistemas de apoyo para la toma de decisiones, alertas tempranas y entrenamiento de sistemas expertos, entre otros. Cuando la fuente de lo datos se origina en registros clínicos electrónicos surge el concepto de fenotipo computacional. En algunos casos, la obtención de los datos clínicos puede ser difícil por barreras de acceso, escasez de fuentes de información estructuradas uniformemente, limitaciones en la calidad de los datos y costos elevados, entre otros ^2^. Además, se enfrenta el reto de modelar eventos de baja frecuencia, donde los datos de un número limitado de instituciones pueden no ser representativos de la población general.

Ante esto, el uso de datos sintéticos puede contribuir a la disponibilidad de datos clínicos que de otra forma podrían ser difíciles obtener. La generación de datos sintéticos puede ayudar a generar gran cantidad de datos que, una vez ajustados, permiten optimizar los procesos de simulación de poblaciones y la interpretación de hallazgos clínicos de forma rápida, a bajo costo y de manera flexible ^3^. En este contexto, se estaría generando un fenotipo computacional que utiliza algoritmos aplicados a datos clínicos para derivar patrones de grupos de pacientes con manifestaciones clínicas de interés ^4^.

En los objetivos de desarrollo sostenible se encuentra priorizada la reducción en un 75 % de la mortalidad materna y perinatal, considerada como un indicador clave del estado de desarrollo de una sociedad; sin embargo, continúa siendo un desafío persistente, especialmente en los países en desarrollo ^5^^-^^7^. Esto se evidencia en la razón de la mortalidad materna; por ejemplo, en el 2019, en Chile, murieron 21,8 mujeres por cada 100.000 nacimientos vivos, mientras que en Haití murieron 326,2 mujeres por la misma cantidad de nacimientos ^8^. En Colombia, la razón de mortalidad materna se ha estimado en 49,0 muertes de mujeres por cada 100.000 nacidos vivos y el objetivo es reducirla a 32 muertes por cada 100.000 nacidos vivos para el 2026 según el plan decenal ^9^.

Para lograr este objetivo se debe tener presente que el proceso gestacional trae consigo riesgos e involucra una diversidad de condiciones fisiológicas, morbilidades y complicaciones graves que pueden llevar a la muerte o a consecuencias serias para la madre y el producto del embarazo ^10^. Las principales causas de mortalidad materna y perinatal identificadas son los episodios de sangrado y las hemorragias, los trastornos hipertensivos asociados a la gestación, las infecciones, las complicaciones del parto y los abortos ^11^. Es por ello que, en el contexto de la gestación, existen estudios en los que se han utilizado modelos de generación de datos sintéticos aplicados a la salud con la finalidad de contribuir al desarrollo de *software* y pruebas, procesos de entrenamiento en salud, investigación en salud pública, predicción y simulación en investigación, etc. ^12^. Además, estas investigaciones indican las ayudas disponibles para el uso de plataformas y fuentes de datos sintéticos junto con los modelos disponibles, incluso para el caso específico de gestantes.

Para la generación de datos sintéticos existen diversas herramientas, una de ellas es Synthea™, que se caracteriza por su distribución gratuita de código abierto para la generación de datos sintéticos ^13^. Esta plataforma genera historias clínicas longitudinales basadas en datos demográficos, epidemiológicos, condiciones médicas y tratamientos; cada registro sintético contiene un conjunto de datos clínicos estructurados en torno a consultas, citas o atenciones (*encounters*), que permite representar situaciones típicas. Asimismo, Synthea*™* cuenta con un módulo de embarazo.

Sin embargo, la implementación de este modelo de gestación para Colombia está limitado por la demografía del país y por diferencias en los protocolos del sistema de salud y las redes de atención. Por tal motivo, este estudio tuvo como objetivo formular un modelo de generación de datos sintéticos para el contexto gestacional colombiano y normalizar dichos datos al modelo de datos común (*Common Data Model*, CDM) de la *Observational Medical Outcomes Partnership* (OMOP) con el fin de superar las dificultades encontradas en los registros clínicos electrónicos tradicionales, en los cuales los campos para el inicio, fin y edad gestacional al momento del nacimiento no existen de forma congruente en las historias clínicas ^14^^,^^15^.

## Materiales y métodos

Se realizó un estudio de tipo reporte de caso centrado en la construcción de un modelo de generación de datos sintéticos en torno al curso clínico de las gestaciones en Colombia. El proceso de modelado se basó en principios de dinámica poblacional y se desarrolló en tres etapas o fases: 1) diseño del modelo de atención; 2) configuración y diagramación del modelo en Synthea™, y 3) generación de los datos sintéticos y su conversión al modelo común de datos.

### Diseño del modelo de atención

En esta etapa participaron un médico general, un médico epidemiólogo y un ginecoobstetra para la revisión de las guías de manejo y la literatura científica ^16^. Se desarrolló un diagrama de flujo de la evaluación del embarazo en Colombia con las complicaciones más frecuentes (trastornos hipertensivos asociados al embarazo, aborto, embarazo ectópico, hemorragias del tercer trimestre y hemorragia posparto) y sus respectivas estimaciones de prevalencias y probabilidades de ocurrencia. según la literatura o según la experiencia para los casos en los que no se encontraron datos (https://github.com/evertorres/maternal-health-synthetic-data-omop).

### Configuración inicial y diagramación del modelo en Synthea™

Si bien Synthea™ ha sido desarrollado principalmente para el mercado de Estados Unidos y sus actualizaciones han permitido generar datos sobre otras poblaciones internacionales (Synthea International) ^17^, no existe disponibilidad de proveedores de salud u hospitales, datos demográficos, modelos de atención propios y costos para Colombia. Por esta razón, se formuló una nueva configuración adaptada a la realidad colombiana. Para la configuración de las variables demográficas se utilizaron los datos del censo poblacional del DANE del 2018 ^18^; la ubicación geográfica de los municipios se obtuvo de los códigos de la división política-administrativa (DIVIPOLA) ^19^ y la información de las instituciones de salud se obtuvo del sitio de datos abiertos del gobierno colombiano ^20^. No se configuraron los costos para Colombia por la complejidad del modelo de salud.

En la configuración de los elementos del modelo se usó la herramienta *Module Builder* disponible en Synthea™ y se generó un archivo formato JSON según la arquitectura de alto nivel de la herramienta ^21^. La codificación utilizada para cada uno de los estados fue validada entre un informático clínico y un ingeniero biomédico mediante SNOMED CT, versión 2024.03.01. Para la clasificación de los procedimientos y los códigos diagnósticos previstos del curso de la atención gestacional, se utilizó el código único de procedimientos en salud y la Clasificación Internacional de Enfermedades, versión 10 (CIE-10).

### Generación de datos sintéticos y su conversión a un modelo común

Dado que Synthea™ genera población que transita por todos los módulos existentes, para la generación de datos sintéticos se desarrolló un módulo adicional para obtener solo pacientes en estado de embarazo para hacer más eficiente el proceso de ajuste del modelo objetivo de este estudio.

De la misma manera, la generación de pacientes se realizó por una sola región, por lo que se configuró un libreto (*script*) en código *bash* para facilitar la generación automática de datos sintéticos para cada uno de los departamentos de Colombia, según las distribuciones poblacionales descritas en el censo del DANE del 2018.

Una vez generado el conjunto de datos sintéticos, se utilizó la herramienta ETL (*extract, transform, load*) de Synthea™ ^22^ para su conversión al modelo común de la OMOP, versión 5.4. Se realizaron ajustes al código para que permitiera registrar la ubicación según la división política de Colombia. El uso del modelo común busca estandarizar la estructura y el contenido de los datos derivados de las observaciones clínicas y permitir análisis eficientes de los mismos, al usar términos comunes para los diferentes sistemas de información y registro de las historias clínicas ^14^.

El proceso de generación de datos fue de carácter cíclico e iterativo. Inicialmente, se realizaron simulaciones con diferentes tamaños muestrales hasta alcanzar la cohorte final de 10.637 mujeres gestantes. En cada iteración se llevó a cabo una revisión cualitativa de casos individuales seleccionados por conveniencia para verificar la coherencia clínica de los registros generados. Esta revisión incluyó la identificación de inconsistencias evidentes (por ejemplo, gestaciones en sujetos masculinos o secuencias clínicas incompatibles con el curso de la gestación) y la evaluación de su correspondencia general con el juicio clínico de los especialistas en obstetricia.

Además, se compararon cualitativamente las frecuencias de los principales desenlaces clínicos simulados con las estimaciones disponibles en la literatura nacional e internacional (cuadro 1). Los ajustes al módulo de embarazo, al filtro de pacientes gestantes y a la transformación ETL fueron efectuados en cada etapa hasta que el equipo investigador, por consenso, consideró que el modelo reproducía de manera satisfactoria el contexto clínico colombiano.

**Cuadro 1.** Resumen de desenlaces sus frecuencias de generación y contraste con las frecuencias estimadas en la fase de diseño

Código SNOMED-CT	Condición clínica	Eventos observados n (%)	Reporte en la literatura (%)	Diferencia frente a lo reportado (%)*	Referencias
34801009	Embarazo ectópico	218 (2,1)	2	0,1	^23^
19169002	Aborto en el primer trimestre	1.714 (16,1)	10-20	6,1 a -4,0	^24^^,^^25^
85116003	Aborto en el segundo trimestre	636 (6,0)	5	1,0	^26^
48194001	Hipertensión inducida por el embarazo	107 (1,0)	2-8	-1,0 a -7,0	^24^
38341003	Hipertensión arterial, trastornos	604 (5,7)	2-8	3,7 a -2,3	^24^
37618003	Complicación de hipertensión arterial; razones para cuidados en el embarazo	92 (0,9)	2-8	-1,1 a -7,1	^24^
398254007	Preeclampsia	802 (7,5)	2-8	5,5 a -0,5	^24^
198992004	Eclampsia	445 (4,2)	2-8	2,2 a -3,8	^24^
40801000119106	Diabetes mellitus gestacional y complicaciones del embarazo	696 (6,5)	2-5	4,5 a 1,5	^27^
22033007	Restricción del crecimiento fetal	30 (0,3)	8-10	-7,7 a -9,7	^28^
367494004	Recién nacido prematuro	603 (5,6)	7,2	-1,6	^29^
44223004	Ruptura prematura de membranas	0 (0,0)	8-10	-8,0 a -0,0	^30^
106004004	Complicaciones hemorrágicas del embarazo	172 (1,6)	4	-2,4	^31^
47821001	Hemorragia posparto	1.443 (13,6)	4-6	9,6 a 7,6	^32^

La columna “Diferencia frente a lo reportado (%)” presenta la distancia del valor observado respecto al límite inferior y al límite superior del rango documentado en la literatura. Los valores positivos indican una distancia mayor a la de referencia; los valores negativos indican una menor distancia. Cuando la literatura consultada ofrece un valor puntual, la diferencia se calcula directamente.

### Consideraciones éticas

El proyecto del cual se deriva este artículo fue evaluado por el Comité de Ética de Investigación en Salud de la Universidad Pontificia Bolivariana y fue aprobado en el Acta 7 de 2024.

## Resultados

### Diseño del modelo de atención

El modelo de datos sintéticos construido incluyó los siguientes desenlaces clínicos en los controles prenatales, los cuales se agrupan usualmente por trimestre gestacional: aborto y embarazo ectópico (primer trimestre); trastornos hipertensivos como hipertensión crónica, preeclampsia y eclampsia (segundo trimestre); placenta previa, *abruptio* de placenta (tercer trimestre); y hemorragia posparto (posparto inmediato). Los datos se encuentran disponibles para descarga en Kaggle ^33^.

Acerca de los elementos del modelo y su implementación en una máquina de estado, los principales estados de transición intervenidos fueron: consultas, citas o atenciones (*encounters*) o momentos de la atención de las mujeres gestantes (controles prenatales); los procedimientos de laboratorio o prácticas quirúrgicas derivadas de los posibles desenlaces previstos, y, finalmente, los desenlaces clínicos o condiciones de salud que fueron codificadas en SNOMED-CT.

**Figura 1.**
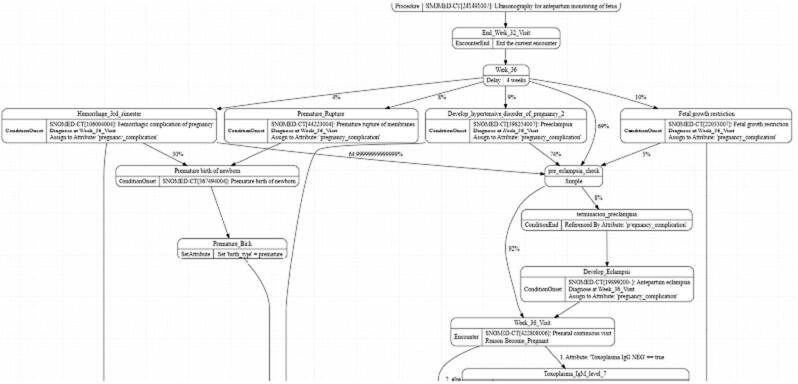
Fragmento derivado del esquema del modelo gestacional en Synthea™

La figura 1 contiene un fragmento del modelo gestacional desarrollado en Synthea™, el cual también puede ser consultado en el repositorio de GitHub (https://github.com/evertorres/maternal-health-synthetic-data-omop).

Disponible en: https://github.com/evertorres/matemal-health-synthetic-data-omop

Como se evidencia en el cuadro 2, al comparar el modelo de gestación disponible en Synthea™ con el modelo desarrollado en este estudio, se logró reducir la cantidad de elementos en un 12,8 % en comparación con el modelo predecesor en Synthea™, pasó de 179 a 156 estados de transición.

**Cuadro 2.** Resumen comparativo de los elementos presentes en los dos modelos de datos utilizados

Tipo de elemento en el modelo de atención gestacional*	Modelo de gestación Synthea™*	Propuesta de nuevo modelo gestacional según el proceso de atención en Colombia
Estados	179	156
Consultas, citas o atenciones (*encounters*)	18	16
Procedimientos	75	72
Condiciones (desenlaces clínicos)	13	16
Desenlace tipo de muerte	0	1

* Reportados por Synthea™ ^34^

No se encontraron mayores diferencias en la cantidad de consultas, citas, atenciones (*encounters*) y procedimientos entre ambos modelos de gestación; sin embargo, el modelo propuesto en este estudio contiene mayor número de desenlaces clínicos. Esta decisión se debió a su asociación clínica a la morbimortalidad materna y fetal, y a su priorización en las guías de práctica clínica para la prevención, la detección temprana y el tratamiento de las complicaciones del embarazo, parto o puerperio ^16^.

El modelo no incluyó condiciones como embarazo anembrionado o antecedentes de aborto previo, dado que usualmente no se asocian a complicaciones mayores o mortalidad materna. Contiene procedimientos de relevancia para el seguimiento y la atención prenatal como la administración de vacunas (tétano, influenza y tos ferina), según la guía de práctica clínica colombiana.

La mayoría de las probabilidades asignadas a las transiciones entre los nodos y los estados del modelo se definieron con ayuda de reportes en la literatura local o mundial; en ausencia de dicha información, la probabilidad se definió según el juicio clínico del equipo que contaba con la participación de un especialista en ginecoobstetricia.

### Conjunto de datos generados

**Figura 2.**
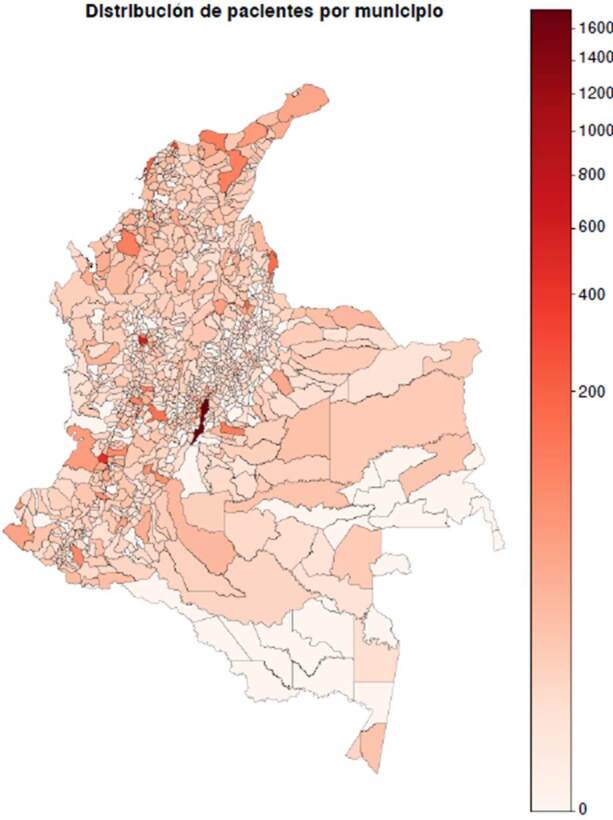
Distribución geográfica por municipio de las mujeres gestantes con el modelo diseñado

Como parte del resultado del modelo y de las pruebas de su funcionamiento, se generó un conjunto de datos de 10.637 pacientes de toda Colombia. La figura 2 ilustra la distribución de pacientes por municipio de nacimiento conservando la proporcionalidad del último censo poblacional. Además, se logró una distribución porcentual por enfermedades que se comparó con los parámetros de configuración iniciales (cuadro 1).

### Aprendizajes de la configuración del modelo en Synthea™

Durante el proceso de prueba y ajuste del modelo en Synthea™, se llevaron a cabo varias iteraciones para la generación de los datos sintéticos. Este ejercicio fue fundamental para evaluar y calibrar el comportamiento del modelo, lo cual permitió analizar sus características clínicas y demográficas, y hacer los ajustes necesarios para mejorar su representatividad en el contexto colombiano.

A pesar de las modificaciones implementadas, algunas de las limitaciones presentes en el módulo original de embarazo de Synthea™ no pudieron ser completamente resueltas. Entre ellas, se destaca que, a pesar de haber incluido algunos estados de transición hacia la mortalidad materna, no se generaron datos con este resultado, lo que impide modelar con precisión uno de los eventos más críticos en salud materna y que, a diferencia del modelo original, sí puede ser representativo en nuestra región. Asimismo, el modelo no contempla el impacto del consumo de sustancias como el tabaco o el alcohol durante la gestación, un factor relevante en los estudios epidemiológicos y en la evaluación de los riesgos perinatales. Otra limitación importante se relaciona con la interacción entre los distintos módulos de Synthea™, ya que la combinación de múltiples condiciones puede generar comportamientos inesperados, como se menciona en la documentación oficial de la herramienta. De la misma manera, la generación de datos transita por todos los módulos que ya están previamente diseñados en Synthea™ y que aún no han sido ajustados al contexto local. En este sentido, la simulación hecha no logra capturar con fidelidad el comportamiento de la población colombiana.

El diseño del modelo se estructuró considerando el curso del embarazo semana a semana, de modo que las condiciones agregadas aparecen en momentos específicos del período gestacional. Sin embargo, los resultados obtenidos presentan un elevado grado de determinismo, lo que reduce la variabilidad esperada en un conjunto de datos reales. En consecuencia, será necesario incorporar distribuciones probabilísticas dinámicas para evitar que todas las mujeres gestantes sigan patrones idénticos, lo que permitirá una mayor diversidad en la generación de situaciones clínicas y mejorará la capacidad del modelo para representar situaciones reales con mayor precisión.

Uno de los logros más relevantes del desarrollo de este trabajo fue la transformación de los datos generados al modelo de datos común de la OMOP, lo que abre la posibilidad de una mayor adopción internacional de la base de datos y facilita su interoperabilidad con otros sistemas basados en este estándar. No obstante, este proceso de conversión también presentó varios desafíos. La herramienta ETL-Synthea solo permitía el mapeo de ciudades y estados de los Estados Unidos, por lo que fue necesario modificar el código para incluir la conversión de los departamentos y municipios de Colombia. Además, la transformación de datos no contempló algunos elementos clave del modelo original, como la clasificación detallada de los consultas, citas o atenciones clínicas (*encounters*) con el personal de salud y los resultados cualitativos de mediciones y observaciones. Esta limitación resultó en una pérdida significativa de información que podría ser crucial para el análisis detallado de los datos en futuras investigaciones.

Estos hallazgos resaltan la importancia de un proceso iterativo de calibración para adaptar modelos generados con Synthea™ a poblaciones específicas. Asimismo, ponen en evidencia la necesidad de seguir mejorando la conversión de datos hacia formatos estandarizados, como el modelo común de la OMOP, con el fin de maximizar la utilidad de la base de datos en el ámbito de la inteligencia artificial y la ciencia de datos aplicadas a la salud materna.

## Discusión

En la práctica, Colombia carece de un sistema nacional de información en salud que permita la recopilación sistemática de datos clínicos, particularmente de historias clínicas electrónicas. Esta carencia, sumada a la limitada interoperabilidad entre los sistemas y al escaso uso de vocabularios y terminologías estandarizadas, dificulta el acceso a grandes volúmenes de datos clínicos estructurados.

Con el fin de solventar este vacío, este estudio presenta varias fortalezas: en primer lugar, se incorporaron elementos demográficos propios del país, lo cual aporta pertinencia contextual al modelo desarrollado; en segundo lugar, se utilizaron estimadores porcentuales basados en los datos nacionales sobre las principales enfermedades asociadas con el embarazo, especialmente aquellas con mayor vínculo clínico con complicaciones maternas y fetales graves; en tercer lugar, se empleó la herramienta de simulación basada en máquinas de estado Synthea™ y se realizó una comparación entre el modelo propuesto y otro previamente desarrollado en el ámbito de la gestación para los Estados Unidos ^34^; y, finalmente, se integró el modelo de datos común de la OMOP, versión 5.4, lo que contribuye a la estandarización y potencial interoperabilidad del modelo.

El proceso de trabajo se vio enriquecido por la conformación de un equipo investigador multidisciplinario, con experticia clínica, epidemiológica y en informática de la salud. Cabe señalar que los datos de referencia utilizados provienen de diversas fuentes internacionales, por lo que no representan con precisión la epidemiología colombiana. Para lograr una adecuada generalización será necesario contrastar las distribuciones de los resultados con datos clínicos reales.

Entre las principales limitaciones del estudio se encuentra la evaluación del modelo y la validación de los datos sintéticos generados. Aunque en Colombia existe el Sistema de Información de la Protección Social (SISPRO), el acceso a los datos primarios es restringido, lo que impidió realizar validaciones cuantitativas y estadísticas sólidas (*robust*). En consecuencia, la evaluación se basó en la literatura científica, predominantemente agregada, lo que derivó en un enfoque cualitativo.

Durante el diseño y la configuración del modelo, se identificaron tres factores clave: 1) el carácter innovador de aplicar un modelo de datos sintéticos al proceso de atención de la gestación, dado que esta población ha sido escasamente representada en los conjuntos de datos internacionales; 2) la necesidad de mejorar los procesos de evaluación y ajuste de estos modelos antes de su implementación generalizada, y 3) el gran potencial que ofrece el campo emergente de los datos generativos, como los sintéticos, en la investigación en salud.

A partir de la revisión exhaustiva de la literatura, no se identificaron antecedentes relevantes sobre el uso de datos sintéticos en obstetricia, salvo el modelo base incluido en la herramienta Synthea™ ^(34)^, sin evidencia de aplicaciones o validaciones posteriores. El uso de Synthea™ permitió al equipo investigador comprender las limitaciones inherentes de adaptar una herramienta diseñada originalmente para el contexto estadounidense a las condiciones particulares de atención en Colombia (por ejemplo, la demografía municipal y el acceso a los servicios de salud, entre otros).

Respecto a la evaluación hecha mediante la comparación de las medidas de frecuencia entre los datos sintéticos y la literatura, se recomienda una interpretación cautelosa. El modelo se basó en estimadores porcentuales fijos, sin incorporar la variabilidad reportada (intervalos de confianza), lo que puede afectar la estabilidad de los resultados.

El potencial identificado en el uso de datos sintéticos coincide con lo descrito por Rajotte *et al*. ^35^, quienes destacan beneficios como el mayor volumen de datos, la posibilidad de representar poblaciones específicas, la protección de la privacidad de los datos reales y la mayor facilidad para compartir la información entre distintos actores. Asimismo, esta estrategia tiene el potencial de promover la equidad y la inclusión en la investigación en salud, especialmente de poblaciones subrepresentadas, como las mujeres gestantes ^36^.

En el futuro, el modelo de generación de datos sintéticos podría ser utilizado para la creación de gemelos digitales y para el desarrollo de análisis causales y prospectivos ^37^. Esta estrategia representa una innovación en el campo de la salud, con aplicaciones prometedoras en el proceso de atención de la gestación, al mejorar la privacidad, ampliar la diversidad poblacional en los estudios y facilitar el desarrollo de aplicaciones de inteligencia artificial, como la consolidación de fenotipos computacionales de morbilidad y mortalidad materna.

Además, las investigaciones futuras deberían iniciar con la aplicación de pruebas estadísticas básicas que permitan comparar las distribuciones, proporciones y medidas de tendencia central entre los datos reales y los sintéticos, con el fin de garantizar su validez inicial. Posteriormente, sería pertinente implementar validaciones cruzadas de carácter geográfico y temporal, así como planes de control del sobreajuste que fortalezcan la solidez (*robustness*) de los modelos derivados de la inteligencia artificial. Finalmente, una vez cubiertos estos aspectos fundamentales, podría avanzarse hacia la integración de métodos más avanzados de exploración, como el análisis de componentes principales o el análisis factorial de datos mixtos para examinar la estructura interna de los datos y su coherencia con los patrones observados en la literatura o en los datos reales.

En síntesis, este estudio demuestra que la generación de datos sintéticos aplicados a la atención de la gestación en Colombia es factible y constituye un aporte pionero en la región. El modelo desarrollado incorpora estándares internacionales como el modelo de datos común de la OMOP y refleja las principales condiciones obstétricas descritas en la literatura, lo que refuerza su utilidad para la investigación y el desarrollo de aplicaciones en inteligencia artificial.

Como implicación práctica, esta estrategia contribuye a superar la limitada disponibilidad y representatividad de los datos reales en salud materna, lo que favorece la interoperabilidad y las comparaciones a nivel global ^38^. No obstante, persisten desafíos relacionados con la validación cuantitativa, la incorporación de variabilidad probabilística y la confrontación con los datos clínicos reales. Las investigaciones futuras deberán ampliar su alcance a otras enfermedades maternas y perinatales, y fortalecer los procesos de validación para consolidar el papel de los datos sintéticos como herramienta innovadora para apoyar la investigación y el desarrollo tecnológico en salud.

## Archivos suplementarios

Anexo: disponibilidad de datos

Los datos sintéticos generados en el marco de este estudio se encuentran disponibles para su consulta y descarga en el portal Kaggle, en el siguiente enlace: https://www.kaggle.com/datasets/evertorres/maternal-health-incolombia-synthetic-data

Todos los archivos, configuraciones y libretos (*scripts*) utilizados para la generación de los datos sintéticos en Synthea™, así como los procedimientos de transformación al *Common Data Model* (OMOP-CDM), se encuentran documentados y accesibles en el repositorio de GitHub:

https://github.com/evertorres/maternal-health-synthetic-data-omop

Este repositorio incluye instrucciones detalladas que permiten la replicación completa del proceso de generación y transformación de los datos.
